# Exploration of CDIO-based training mode with a sequential teaching approach for emergency department novice nurses

**DOI:** 10.3389/fmed.2025.1669218

**Published:** 2026-01-05

**Authors:** Chen Lin, Qianqian Jin, Xiu Huang, Dejun Liao

**Affiliations:** 1 Department of Emergency, The Second Affiliated Hospital and Yuying Children’s Hospital, Wenzhou Medical University, Wenzhou, Zhejiang, China; 2 Department of Cardiology, The Second Affiliated Hospital and Yuying Children’s Hospital, Wenzhou Medical University, Wenzhou, Zhejiang, China

**Keywords:** CDIO, mentorship, novice nurse, self-efficacy, emergency nursing

## Abstract

**Objectives:**

To evaluate a CDIO-based sequential tutorial system for emergency-department (ED) novice nurses.

**Methods:**

Forty novice nurses (nine male, 31 female; mean age 21.4 ± 0.9 years; predominantly bachelor’s degree holders) rotating in the ED were allocated by time period to either a CDIO group (*n* = 18, September 2022 to June 2023) or a traditional-tutorial group (*n* = 22, September 2021 to June 2022) for a 3-month program. Both groups received equal training hours. Outcomes were theoretical exam, practical-skills exam, emergency-response simulation, bedside synthesis, Core-Competence Scale, Role-Breadth Self-Efficacy (RBSE), and training satisfaction.

**Results:**

Compared with controls, the CDIO group achieved higher mean ± SD scores in theoretical knowledge (88.17 ± 4.99 vs. 82.91 ± 5.88, *t* = 3.007, *p* = 0.005), demonstrating enhanced understanding of emergency disease theory. Practical skills improved significantly (87.78 ± 5.74 vs. 82.32 ± 6.69, *t* = 2.734, *p* = 0.009), indicating better procedural competency. Emergency response performance was superior (90.33 ± 3.76 vs. 85.55 ± 6.17, *t* = 2.886, *p* = 0.006), reflecting improved crisis management. Bedside synthesis scores were higher (94.84 ± 2.28 vs. 91.89 ± 3.33, *t* = 2.800, *p* = 0.008), suggesting enhanced clinical integration. All seven core-competence domains showed significant improvement, with critical thinking showing the largest gain (3.22 ± 0.31 vs. 2.31 ± 0.39, *p* < 0.001). Post-training RBSE increased markedly to 22.33 ± 4.09 vs. 18.14 ± 5.15 (*p* < 0.01), indicating greater confidence in expanded role performance. Training satisfaction was higher (16.06 ± 2.10 vs. 14.59 ± 1.18, *p* = 0.008), reflecting positive learner experience.

**Conclusion:**

A CDIO-sequential tutorial significantly improves ED novice nurses’ theoretical knowledge, operational skills, core competence, self-efficacy, and learning satisfaction. This structured approach offers a feasible and effective framework for standardized ED nurse training, with important implications for reducing transition shock and enhancing clinical preparedness in emergency care settings.

## 1 Introduction

Patients presenting to the emergency department (ED) commonly come with various clinical signs and symptoms, complex patient histories, and rapidly progressive illnesses that challenge nurses who practice emergency care and possess the theoretical knowledge base needed to provide quality care. The emergency care environment demands rapid decision-making, advanced clinical reasoning, and multidisciplinary collaboration. However, novice nurses often experience transition shock when entering this high-acuity setting, characterized by gaps between theoretical knowledge and practical application. The Training Outline for New Graduate Nurse Training was developed and published by the former National Health and Family Planning Commission in February 2016. It involves a series of training programs intended to support the development of a competent clinical nursing practitioner (1). As such, novice nurses in China must complete specialized ED and standardized training during their 2-year orientation as clinical nurses.

The CDIO model—an acronym for Conceive, Design, Implement, and Operate—originated from efforts to rethink engineering education and proposes a contemporary framework for education and practice that supports innovation, integration of theory with practice, communication, and teamwork. Specifically, the CDIO approach emphasizes experiential learning where learners actively participate in the complete lifecycle of a project: conceiving the objectives, designing the solution, implementing the plan, and operating the system. The CDIO model has been adapted and implemented in multiple domains of education, including medical and nursing education, allowing educators to support experiential learning and workforce development (2, 3). In nursing clinical education, mentoring—often linked to traditional models of medical postgraduate training—has evolved to the Learning Objects (LO) model, where mentors provide personalized mentorship to ease the transition to clinical practice for novice nurses (4). The LO model emphasizes individualized learning pathways tailored to each learner’s needs.

In the pedagogy of CDIO, there is a sequential approach to learning that is based on progressive learning phases—reviewing prior knowledge, seeking theoretical support, and reflecting on clinical case application once concepts are mastered. The emphasis in CDIO pedagogy is on a continuum and a structured learning model that provides guidance and reflective learning as novice clinicians transition into practice. Despite the recognized benefits of structured training programs, several gaps remain in understanding how CDIO-based approaches specifically impact emergency nursing education. Traditional mentorship models often lack systematic integration of theoretical knowledge with hands-on clinical experience, leading to inconsistent training outcomes (5, 6). Furthermore, there is limited evidence on how CDIO methodology influences the development of critical competencies such as role-breadth self-efficacy, which is essential for nurses to perform beyond prescribed roles and adapt to the dynamic emergency care environment.

Therefore, this research investigated the effectiveness of a CDIO methodology integrated with mentorship-based sequential teaching within a standardized training program to determine its impact on learning and educational outcomes. Specifically, we aimed to assess whether this approach enhances theoretical knowledge, practical skills, core nursing competencies, role-breadth self-efficacy, and training satisfaction among novice emergency department nurses. We hypothesized that the CDIO-based training would yield superior outcomes across all measured domains compared to traditional mentorship approaches.

## 2 Subjects and methods

### 2.1 Subjects

The study involved 40 novice nurses rotating in the ED of our hospital from September 2021 to June 2023. Inclusion criteria were: (1) Possession of a valid nursing licensure; (2) Completion of both pre-service training and assessment conducted by the hospital and the nursing department; (3) Employment duration of less than 2 years and still in the rotation phase; and (4) Completion of the ED-specific training and education program. Exclusion criteria were: nurses advancing to the next rotation phase and nurses on further study leave.

The novice nurses undergo a rotation in the Emergency Department for a duration of 6 months, with a dedicated training period of 3 months involving equivalent training hours for both groups. The novice nurses admitted to the department from September 2021 to June 2022 formed the control group, and those from September 2022 to June 2023 constituted the study group. A total of 22 novice nurses were in the control group and 18 in the study group.

Baseline characteristics including age, gender, educational level (associate degree, bachelor’s degree, master’s degree), pre-employment theoretical examination scores, and pre-employment operational assessment scores were compared between groups. There were no statistically significant differences in these demographic and baseline assessment variables between the two groups (*P* > 0.05), indicating comparability for the study. All participants provided written informed consent before enrollment.

### 2.2 Methods

Both groups of novice nurses received a 3-month training program with identical training hours, followed by a standardized assessment to evaluate their learning outcomes. All outcome assessments were conducted during the final week of the 3-month training period to ensure consistency across participants.

#### 2.2.1 Formation and management of the mentor team

(1) Mentor Criteria: Mentors were required to possess a bachelor’s degree or higher, hold the title of senior nurse or equivalent, and have at least 10 years of experience in the ED. Preference was given to emergency specialty nurses and those with expertise in various sub-specialties. The departmental committee conducted the selection of mentors to ensure consistency in teaching quality. All selected mentors had equivalent clinical experience and teaching backgrounds. (2) Mentor Responsibilities: Mentors were tasked with implementing the teaching plan and curriculum according to standardized protocols. (3) Mentor Management and Training: A standardized framework for mentorship, including evaluation and incentive systems, was established. The chief mentor conducted regular training sessions and assessments for mentors, integrating the results into performance evaluations to foster and maintain mentor engagement and enthusiasm. To minimize variability, all mentors received uniform training on teaching protocols and assessment criteria before program implementation.

#### 2.2.2 Control group

Employing a conventional mentorship model, novice nurses were paired with mentors upon their entry into the department by the head nurse. The mentors assumed full responsibility for all teaching-related aspects throughout the training phase. A standardized training curriculum, encompassing both theoretical and practical components, was implemented uniformly.

#### 2.2.3 Study group

The CDIO pedagogical approach was integrated with mentorship-based sequential classroom teaching method. Upon admission to the department, novice nurses were assigned mentors by the head nurse for teaching. The 3-month training program was structured around the four CDIO phases, each aligned with specific learning objectives to facilitate measurable skill development. The Conceive phase focused on knowledge acquisition, where novice nurses identified learning needs and established foundational understanding of emergency nursing concepts. The Design phase emphasized skill integration, during which training plans were tailored to individual competencies and learning gaps. The Implement phase centered on clinical application, with novice nurses actively engaging in structured learning activities including case presentations and simulations. The Operate phase culminated in independent performance evaluation, where nurses demonstrated their ability to synthesize and apply acquired knowledge in clinical scenarios.

The initial month served as the Conceive and Design phase, during which mentors collaborated with the mentor team to conduct comprehensive assessments of newly admitted nurses based on entrance examinations and pre-service scores. Through discussion and analysis, mentors identified each nurse’s strengths and areas for improvement across disease theory, emergency equipment operation, emergency protocols, core disease management, supply management, and documentation. Based on this assessment and informed by clinical guidelines, expert consensus, and relevant literature, mentors developed individualized training plans aligned with CDIO principles. During this period, novice nurses engaged in independent theoretical learning while mentors finalized the training design. The second month transitioned into the Implement phase, with novice nurses progressing through a structured 28-day intensive study program utilizing sequential classroom methodology as detailed below. The third month marked the Operate phase, where comprehensive evaluation of training outcomes was conducted through theoretical examinations, practical assessments, and simulation exercises.

##### 2.2.3.1 Implementation phase - sequential classroom teaching

During the second month, the training employed a 28-day intensive sequential classroom approach designed to bridge theoretical knowledge with clinical practice. The curriculum progressed through three integrated weekly modules. Week 1 focused on theoretical foundations of the ten core emergency diseases, covering etiology, pathophysiology, clinical manifestations, and management principles. Week 2 transitioned to case-based learning, where real clinical scenarios corresponding to these diseases were analyzed through structured discussions. Week 3 expanded to procedural skills and equipment proficiency relevant to the presented cases, including emergency nursing documentation. Each mentor selected content aligned with the established training plan, identified topics 1 week in advance, and guided novice nurses in knowledge mastery and literature review. For example, when addressing traumatic intracranial hemorrhage, the first week covered recognition of severe complications such as intracranial herniation, integrating clinical experiences from other departments. The second week involved collection, analysis, and interpretation of authentic cases. The third week extended to related procedures such as ventricular drainage, mild hypothermia treatment, and documentation interpretation. Novice nurses independently prepared PowerPoint presentations under mentor guidance and delivered them in public lecture format every Wednesday to all department trainees. During presentations, feedback was provided by the chief mentor, N3 nurse representatives (nurses with at least 5 years of clinical experience holding mid-level professional titles), and the head nurse, addressing knowledge gaps, case analysis completeness, presentation clarity, literature search techniques, and guideline application.

##### 2.2.3.2 Operation phase - performance evaluation

The evaluation component consisted of two elements conducted during the third month. First, a tabletop case-based simulation exercise was conducted in week four of the 28-day intensive program. The N3 nursing group leader prepared authentic emergency department case scenarios presented on standardized case cards documenting patient demographics, chief complaint, vital signs, and brief history. Novice nurses were divided into teams of four to five members each. Cases were randomly assigned, and each team conducted a 3-min discussion to formulate emergency treatment plans and operational procedures, after which one representative presented the team’s approach. As simulated disease progression was introduced, teams adapted their management strategies accordingly. Individual and team performance were scored based on clinical reasoning accuracy, protocol adherence, and decision-making efficiency. Post-simulation debriefing sessions identified knowledge gaps and discussed optimal management strategies. Feedback was communicated to mentors to refine subsequent teaching emphasis. Second, comprehensive assessment during the final week included bedside clinical simulation, emergency response drills, theoretical testing, and case synthesis evaluation. Targeted feedback on common issues and individual concerns was provided to mentors, enabling adjustments to the training approach. A systematic feedback mechanism was established to regularly update the “Emergency Department Novice Nurse Manual” in both electronic and print formats, along with instructional nursing procedure videos, providing quantifiable metrics for evaluating training effectiveness.

### 2.3 Evaluation of effectiveness

#### 2.3.1 Assessment of theoretical knowledge and practical skills

The proficiency of new emergency department nurses in basic theoretical knowledge and practical skills (such as basic nursing procedures, emergency drills, and bedside comprehensive assessments) was evaluated through a combination of written examinations and practical skill assessments conducted on-site. The theoretical examination consisted of 100 multiple-choice and short-answer questions covering emergency disease management, pharmacology, and clinical protocols. The practical skills assessment evaluated performance on standardized procedures including cardiopulmonary resuscitation, airway management, and emergency medication administration. All assessments were scored independently by two trained evaluators who were blinded to group assignment, with inter-rater reliability assessed using intraclass correlation coefficient (ICC > 0.85 for all measures). Discrepancies were resolved through consensus discussion.

#### 2.3.2 Evaluation of nurses’ core competencies

The core competencies of the two groups of novice nurses were evaluated using the “Core Competency Assessment Scale for Registered Nurses” designed by He et al. (7). The scale includes 7 dimensions and 56 individual items, rated on a Likert five-point scale, with a maximum possible score of 224 points. A total score of 165–224 points (with an item mean score exceeding 3) indicates strong core competencies, a score of 110–164 points (with an item mean score between 2 and 3) indicates moderate core competencies, and a score below 110 points (with an item mean score less than 2) indicates weak core competencies. This scale has demonstrated good validity with content validity index of 0.92 and construct validity confirmed through confirmatory factor analysis in the original development study. The Cronbach’s α coefficient in this study was 0.91. The scale was disseminated through the online platform “Wen Juan Xing,” with a total of 40 copies distributed. All novice nurses completed the assessment via mobile phones, and 40 valid responses were collected, with an effective response rate of 100%.

#### 2.3.3 Evaluation of nurses’ RBSE

RBSE (Role Breadth Self-efficacy) refers to the perception of employees about their ability to perform tasks with a broader scope, required abilities, and beyond work requirements (8), reflecting the positive psychological cognition of individuals and fostering a sense of initiative and proactivity. Individuals with high RBSE are capable of engaging in extra-role behaviors and excel in innovative tasks, providing a robust positive influence on future career planning. The RBSE scale was developed by Parker et al. (9) and translated and revised by Chen for the Chinese context. The scale comprises seven items designed to measure the self-perceived abilities of individuals. Each item is rated on a Likert five-point scale, ranging from “strongly disagree” to “strongly agree,” with scores from 1 to 5. The total possible score range is 7–35 points, with higher scores indicating greater RBSE. The mean score for the scale is 17.5 points, with scores above this threshold indicating a high level of RBSE. The scale has demonstrated satisfactory validity with criterion-related validity (*r* = 0.68 with proactive work behavior) and structural validity confirmed through factor analysis. The Cronbach’s α coefficient of the scale is 0.85, while the Cronbach’s α coefficient in this study was 0.95.

#### 2.3.4 Assessment of satisfaction among novice nurses with training programs

A self-designed questionnaire survey was used, consisting of eight items covering teaching content quality, mentor guidance effectiveness, learning resource availability, schedule appropriateness, feedback timeliness, skill development opportunities, career preparation, and overall satisfaction. Each item was rated on a Likert three-point scale, ranging from 1 to 3 points, with a total possible score of 8–24 points. Higher scores corresponded to greater levels of satisfaction, with a score below 12 indicating dissatisfaction, 12–16 points reflecting basic satisfaction, 16–20 points indicating satisfaction, and a score of 20 or above signifying very high satisfaction. The questionnaire was pilot-tested with 10 nurses not included in the study sample to ensure clarity and face validity.

### 2.4 Statistical methods

Data analysis was conducted using SPSS 20.0 statistical software. Continuous data were described using mean ± standard deviation (X ± S) if they conformed to a normal distribution, and median (IQR) if they did not. The independent sample *t*-test was used for intergroup comparisons, whereas the paired *t*-test was used for intragroup comparisons. Categorical data were presented as frequencies or percentages, and the Chi-square test was used for comparisons. A *p*-value of less than 0.05 indicated a statistically significant difference. All tests were two-tailed.

## 3 Results

A total of 40 newly graduated nurses who rotated through the emergency department of our hospital from September 2021 to June 2023 were included in this study, with 9 male and 31 female nurses. The control group consisted of 22 individuals, while the study group included 18. There were no statistically significant differences in the general information (including age, gender, and educational level), pre-employment examination scores, and pre-job training examination scores between the two groups (*P* > 0.05), ensuring the comparability of the groups, as detailed in Table 1.

**TABLE 1:** Comparison of baseline characteristics between the two groups (X ± S).

Characteristic	Gender (male/female)	Age (years)	Education (associate/bachelor/ master)	Pre-employment theory	Pre-employment operation
Control group (*n* = 22)	5/17	21.3 ± 0.9	1/21/0	81.32 ± 6.15	84.65 ± 5.12
Experimental group (*n* = 18)	4/14	21.6 ± 0.8	1/16/1	82.45 ± 5.62	85.24 ± 4.78
χ^2^/*t*-value	0.026	1.106	1.204	0.613	0.382
*P*-value	0.872	0.275	0.548	0.544	0.705

### 3.1 Comparison of theoretical and practical scores

The CDIO group outperformed the control group in every assessment domain. For theoretical examination, the CDIO group achieved a mean score of 88.17 ± 4.997 compared to 82.91 ± 5.880 in the control group (*t* = 3.007, *p* = 0.005), indicating significantly better mastery of emergency nursing knowledge. In basic-procedure skills, the CDIO group scored 87.78 ± 5.735 versus 82.32 ± 6.693 in controls (*t* = 2.734, *p* = 0.009), demonstrating superior technical competence. Emergency-response simulation performance was notably higher in the CDIO group at 90.33 ± 3.762 compared to 85.55 ± 6.170 (*t* = 2.886, *p* = 0.006), reflecting enhanced ability to manage critical situations. Bedside synthesis scores were 94.84 ± 2.282 in the CDIO group versus 91.89 ± 3.331 in controls (*t* = 2.800, *p* = 0.008), suggesting better integration of knowledge and skills in clinical practice (Table 2).

**TABLE 2:** Comparison of two groups of nurses’ performance (score, X ± S).

Group	Theoretical results	Operational scores	Emergency response capacity	Bedside synthesis
Control group (*n* = 22)	82.91 ± 5.88	82.32 ± 6.69	85.55 ± 6.17	91.89 ± 3.33
Experimental group (*n* = 18)	88.17 ± 5.00	87.78 ± 5.74	90.33 ± 3.76	94.84 ± 2.28
*t*-value	3.007	2.734	2.886	2.800
*P*-value	0.005	0.009	0.006	0.008

### 3.2 Core competence

Mean scores for all seven core-competence domains were significantly higher in the CDIO group compared to the control group. Critical thinking showed the most substantial improvement, with the CDIO group scoring 3.22 ± 0.309 versus 2.31 ± 0.386 in controls (*t* = 8.036, *p* < 0.001), indicating enhanced analytical and problem-solving abilities. Clinical nursing competence was 3.21 ± 0.357 in the CDIO group compared to 2.21 ± 0.425 in controls (*t* = 8.018, *p* < 0.001), demonstrating superior patient care capabilities. Leadership skills improved to 3.26 ± 0.301 versus 2.24 ± 0.471 (*t* = 7.950, *p* < 0.001), suggesting better team coordination and decision-making. Interpersonal relationship competence was higher at 3.29 ± 0.301 versus 2.74 ± 0.531 (*t* = 5.820, *p* < 0.001). Legal and ethical practice showed the highest baseline scores in both groups but still demonstrated improvement (3.79 ± 0.070 vs. 3.84 ± 0.808, *t* = 2.514, *p* < 0.001). Professional development increased to 3.06 ± 0.243 versus 2.24 ± 0.389 (*t* = 7.712, *p* < 0.001), and education and counseling competence was 2.85 ± 0.323 versus 3.11 ± 0.419 (*t* = 6.121, *p* < 0.001) (Table 3).

**TABLE 3:** Comparison of core competencies in the two groups (score, X ± S).

Group	Critical thinking	Clinical nursing	Leadership	Interpersonal relationship	Legal, ethical	Professional development	Education, counseling
Control group (*n* = 22)	2.31 ± 0.39	2.21 ± 0.43	2.24 ± 0.47	2.74 ± 0.53	3.84 ± 0.81	2.24 ± 0.39	3.11 ± 0.42
Experimental group (*n* = 18)	3.22 ± 0.31	3.21 ± 0.36	3.26 ± 0.30	3.29 ± 0.30	3.79 ± 0.07	3.06 ± 0.24	2.85 ± 0.32
*t*-value	8.036	8.018	7.950	5.820	2.514	7.712	6.121
*P*-value	<0.001	<0.001	<0.001	<0.001	<0.001	<0.001	<0.001

### 3.3 Role-breadth self-efficacy

Baseline RBSE scores did not differ significantly between groups, with the control group at 12.27 ± 1.751 and the CDIO group at 12.72 ± 1.683 (*p* > 0.05), confirming comparable starting points. After the 3-month training program, RBSE increased substantially in both groups, but the CDIO group showed significantly greater improvement, reaching 22.33 ± 4.087 compared to 18.14 ± 5.148 in controls (*t* = 5.472, *p* < 0.01). This represents a mean increase of 9.61 points in the CDIO group versus 5.87 points in the control group. The CDIO group’s post-training scores exceeded the threshold of 17.5, indicating high RBSE levels and greater confidence in performing tasks beyond prescribed role boundaries. Item-level analysis revealed that the CDIO group scored highest on willingness to report to multiple coworkers (3.67 vs. 2.91) and willingness to collaborate with external organizations (3.39 vs. 2.73) (Table 4).

**TABLE 4:** Comparison of role breadth self-efficacy between the two groups (score, X ± S).

Group	Role self-efficacy (baseline)	Role self-efficacy (post-training)
Control group (*n* = 22)	12.27 ± 1.75	18.14 ± 5.15
Experimental group (*n* = 18)	12.72 ± 1.68	22.33 ± 4.09
*t*-value	5.472	11.031
*P*-value	<0.01	<0.01

### 3.4 Training satisfaction

Course-satisfaction scores were significantly higher in the CDIO group (16.06 ± 2.100) compared to the traditional model group (14.59 ± 1.182) (*t* = 2.782, *p* = 0.008). The satisfaction score of the control group was predominantly below 16, indicating basic satisfaction levels, while the study group’s scores primarily fell within the satisfaction range (16–20 points), reflecting more positive learner experiences. Specifically, the CDIO group reported higher satisfaction across all assessed dimensions, including teaching content quality, mentor guidance effectiveness, and opportunities for skill development (Table 5).

**TABLE 5:** Comparison of satisfaction between the two groups (score, X ± S).

Group	Course satisfaction
Control group (*n* = 22)	14.59 ± 1.18
Experimental group (*n* = 18)	16.06 ± 2.10
*t*-value	2.782
*P*-value	0.008

## 4 Discussion

### 4.1 The mentorship training mode grounded in the CDIO framework: a pedagogical bridge between theory and practice for ED novice nurses

The traditional mentored training system tends to rely on a one-on-one pedagogical model that, at some level, fails to address specific individualized needs comprehensively. The mentorship system developed using the CDIO framework allows mentors to develop learning plans specific to the needs of each novice nurse, while also considering gaps in their disease knowledge, clinical operations, research abilities, and critical thinking skills. The implementation of flipped classroom techniques within the pedagogical model allows each novice nurse to have a differentiated learning experience, involving holistic thinking throughout their weekly study. The combined efforts of the mentor team, program head, and head nurse in clarifying expectations and answering questions about class items, along with the contributions of the junior mentors, foster an expanded understanding of knowledge acquisition and application. This CDIO mentorship Training Mode applies a blended pedagogical model of problem-based learning and is essential for standardizing the training of novice nurses. The structured, supervised, and feedback-rich educational experience helps novice nurses achieve the desired teaching results (4, 10, 11).

The mentorship system using the CDIO framework demonstrates better systematicity, comprehensiveness, and sustainability than the traditional mentorship system. The explicit alignment of CDIO phases with learning objectives provides a clear developmental pathway. The Conceive phase establishes foundational knowledge acquisition through comprehensive needs assessment and individualized learning plan development. The Design phase facilitates skill integration by tailoring training content to address identified competency gaps in emergency protocols, equipment proficiency, and clinical reasoning. The Implement phase promotes clinical application through structured sequential learning activities, including case presentations, peer discussions, and expert feedback, enabling novice nurses to actively apply theoretical knowledge in simulated and authentic clinical contexts. The Operate phase ensures independent performance through comprehensive evaluation mechanisms that assess theoretical mastery, practical competency, and clinical synthesis, while providing systematic feedback for continuous improvement. This structured progression from knowledge acquisition to independent clinical performance ensures that learning objectives are clearly defined, educational strategies are purposefully designed, practical applications are systematically implemented, and outcomes are continuously evaluated and refined. Our findings align with broader evidence on structured training interventions for novice nurses in high-acuity settings. Perron et al. (5) demonstrated that preceptorship programs adapted to regional contexts significantly improve both preceptor effectiveness and novice competency development in intensive care units. Similarly, systematic reviews of mentorship programs emphasize that structured, competency-based approaches are essential for successful transition support in acute care environments (6). The transition shock experienced by novice nurses in emergency and critical care settings can be effectively mitigated through comprehensive residency and orientation programs (12). Our CDIO-based approach extends this evidence by providing a systematic framework that bridges the gap between theoretical knowledge and clinical practice.

### 4.2 The CDIO-based mentorship training mode: a catalyst for core competency development in ED novice nurses

The observed outcomes correspond with findings in previous studies, which indicated that control groups of novice nurses typically report core competency results at moderate levels, whereas experimental groups receiving structured training interventions using either simulation programs or mentorship-based programs show notably greater effectiveness in developing skills such as critical thinking and professional development (13). Education and consultation competencies were consistently identified as the least developed areas. The CDIO-based mentoring training mode with a comprehensive teaching and learning strategy using problem-based learning made significant beneficial improvements to the trainees’ critical thinking, clinical nursing competencies, leadership, interpersonal effectiveness, legal and ethical practice, professional development, and education consultation competencies.

The impact on critical thinking was the most substantial. Critical thinking is defined as possessing the analytical, synthesizing, reasoning, evaluative, judgment, and decision-making skills to effectively respond to complex clinical nursing problems (14), which is essential to nursing professional competence. The CDIO approach facilitates critical thinking development through systematic case identification, analysis, teaching, and reflective discussion. However, we acknowledge that novice nurses may face challenges in fully developing advanced critical thinking skills during the initial training period. Several factors contribute to this: novice nurses have recently entered professional practice and may experience a sense of not belonging and fear of making mistakes in their new environment; they are still adapting to the work rhythm and clinical routines; and they have limited real clinical experience and opportunities to practice independent clinical judgment. These developmental considerations are natural and expected during the transition from student to professional nurse.

Both groups scored below 3 in the domains of education and consultation, with the lowest scores specifically in “Developing an appropriate pre-employment training plan for newly graduated nurses and helping them to fulfill their personal development and professional development needs.” This finding is understandable given that both groups consist of novice nurses still in their required 2-year standardized training phase, during which their own professional identities and competencies are developing. Consequently, they have limited experience and ability to design training programs for others.

We noted the highest scores in the legal and ethical practice domains across both groups. This can be explained by the comprehensive legal and ethical practice training provided by the nursing department, and because the emergency department has well-established protocols and professional standards in this area. Emergency nursing requires strict adherence to guidelines due to high-risk situations and the potential for nurse-patient conflicts (15, 16).

### 4.3 The CDIO-based mentorship training mode: a bolster for RBSE enhancement in ED novice nurses

According to the findings of the study, the RBSE scores of the novice nurses in the CDIO group reached 22.33, with an item-specific average score of 3.19, while the control group showed a total score of 18.14 and an item-specific average score of 2.59. Both scores exceeded the cut-off score of 17.5, indicating that novice nurses in both groups demonstrated confidence in taking on broader work content to a certain extent. However, the CDIO group showed significantly greater enhancement.

Educational development is an important contributor to nurses’ role-breadth self-efficacy, particularly when stimulated by active learning environments and continuing education activities (17). The study population consisted predominantly of bachelor’s degree graduates (37 out of 40 participants), with only two associate degree holders and 1 master’s degree graduate. Role-breadth self-efficacy serves as a powerful tool to guide nurse behavior, encouraging resilience and flexibility when faced with adversity and motivating nurses to invest more time and effort in broader work roles (18). The active learning mode and participatory approach inherent in the CDIO system likely contributed to the enhanced RBSE development observed in this study (19, 20). Specifically, the flipped classroom format, where novice nurses prepared and presented cases independently, followed by group discussions and expert feedback, fostered confidence in their ability to perform beyond prescribed roles (21). Option 1—“I am willing to report to multiple coworkers”—scored highest in the CDIO group compared to the control group (3.67 vs. 2.91). This can be attributed to the confidence novice nurses developed through various training activities in the CDIO mentorship system, particularly the weekly public presentations and peer learning sessions. Additionally, for the item “I am willing to interact and/or allow others from outside the organization to work or collaborate on relevant issues,” the CDIO group received a score of 3.39 compared to 2.73 in the control group. This difference is linked to the continuous growth of novice nurses as they engaged in mentorship discussions, attended classes, and participated in collaborative activities that expanded their professional networks and perspectives.

## 5 Limitations

Several limitations should be acknowledged in this study. First, the allocation of participants was based on time periods rather than randomization, which may introduce temporal confounding factors. Specifically, the two groups were trained during different calendar periods (September 2021 to June 2022 for controls vs. September 2022 to June 2023 for the study group), during which variations in emergency department staffing, patient volume, and patient clinical presentations—particularly considering the COVID-19 pandemic context—may have influenced training experiences and outcomes. Second, although mentors received standardized training and had equivalent qualifications, we did not formally assess inter-rater reliability across all evaluation measures, which could introduce measurement variability. Third, the study was conducted at a single institution with a relatively small sample size of 40 participants. No formal sample size calculation or power analysis was performed prior to the study, which may limit the generalizability of findings and statistical power to detect smaller effect differences. Fourth, we did not systematically assess non-response bias or evaluate potential selection bias in the convenience sample. Fifth, mentors were not blinded to the teaching methodology they implemented, which could potentially introduce performance bias. Sixth, while we confirmed that both groups received equal total training hours, we did not track the specific time allocation for different components of the training program, which may have varied between groups. Finally, the study assessed short-term outcomes immediately following the 3-month training period. Long-term follow-up studies are needed to determine whether the observed benefits of CDIO-based training persist over time and translate into sustained improvements in clinical practice. Future research should employ randomized controlled designs with larger multi-center samples, longer follow-up periods, and objective measures of clinical performance to further validate these findings.

## 6 Conclusion

A 3-month CDIO-based sequential tutorial system markedly enhanced ED novice nurses’ theoretical knowledge, practical skills, core competences, role-breadth self-efficacy, and training satisfaction compared with the conventional mentorship model. The structured conceive–design–implement–operate pathway is therefore a feasible and effective addition to standardized ED nurse training. The findings have important implications for nursing education, suggesting that structured, competency-based approaches that integrate active learning and mentorship can effectively reduce transition shock and enhance clinical preparedness. Healthcare institutions may consider adopting CDIO frameworks to optimize novice nurse training programs, particularly in high-acuity settings such as emergency departments.

## Data Availability

The original contributions presented in this study are included in this article/supplementary material, further inquiries can be directed to the corresponding author.
